# ‘We would look at the chickens as a source of security’: microenterprise and health in rural Uganda

**DOI:** 10.7189/jogh.15.04074

**Published:** 2025-05-23

**Authors:** Justus Kananura, Bridget FO Burns, Charles Baguma, Rumbidzai C Mushavi, Emily N Satinsky, Allen Kiconco, Elizabeth B Namara, Clare Kamagara, Elijah Musinguzi, Owen Alleluya, Atheendar S Venkataramani, David R Bangsberg, Alexander C Tsai, Bernard Kakuhikire

**Affiliations:** 1 Mbarara University of Science and Technology, Mbarara, Uganda; 2 Beth Israel Deaconess Medical Center, Boston, Massachusetts, USA; 3 Harvard Medical School, Boston, Massachusetts, USA; 4 Vincent Department of Obstetrics and Gynecology, Massachusetts General Hospital, Boston, Massachusetts, USA; 5 Center for Global Health, Massachusetts General Hospital, Boston, Massachusetts, USA; 6 Department of Psychology, University of Southern California, Los Angeles, California, USA; 7 Department of Medical Ethics and Health Policy, Perelman School of Medicine, University of Pennsylvania, Philadelphia, Pennsylvania, USA; 8 College of Health Sciences, VinUniversity, Hanoi, Vietnam; 9 Department of Epidemiology, Harvard T.H. Chan School of Public Health, Boston, Massachusetts, USA

## Abstract

**Background:**

Development interventions may promote sustainable livelihoods among participants via improved income generation, health, education, and quality of life. Within the development literature, microfinance institutions (MFIs) provide individuals with funds and/or start-up capital to develop small businesses. However, the evidence on whether MFIs are successful in ensuring sustainable livelihoods is mixed. In this study, we assessed participants’ perceptions of the barriers and facilitators to a poultry microenterprise intervention, and the impact of the intervention on enabling sustainable livelihoods for the participants, their families, and their community.

**Methods:**

During exit interviews, 30 women who had participated in a poultry microenterprise demonstration project in rural Uganda nine months prior described their experiences in the intervention, including perceived benefits and challenges, and discussed specific factors that impacted their continuity in the project. We analysed the interviews using a content analysis approach.

**Results:**

The participants noted instrumental and interpersonal benefits of the intervention: greater financial security, increased trust from community members, social support, empowerment, and skills-building. Despite these facilitators, challenges precluded some of them from establishing sustainable livelihoods. Pervasive poverty, poultry disease outbreaks, poor spousal/familial support, and challenges in effectively communicating the goal of the intervention stood as barriers to the establishment of sustained poultry businesses. While most participants (n/N = 20/30) reached the final phase of the intervention, only six continued rearing chickens beyond the project.

**Conclusions:**

Barriers and facilitators described by the participants and identified in our analysis bear implications for the design, implementation, and evaluation of microenterprise interventions aimed at providing participants with sustainable livelihoods. Our findings highlight the importance of qualitative research in identifying concerns and informing intervention adaptations.

Sustainability is an economic concept which describes the ability of a group or system to maintain productivity over time [1]. In the international development literature, sustainable development describes the construction of a socioeconomic system that addresses income generation, education, and improvements in health and quality of life within a population [2]. Development interventions that lead to sustainable livelihoods among participants include those resilient to stressors and those maintain target outputs and resources over time [3–8], even after external funding and support are withdrawn.

Microfinance institutions (MFIs) provide individuals with funds and/or start-up capital to develop small businesses and can contribute to the development of sustainable livelihoods and poverty eradication [9]. These initiatives, many of which are targeted at women, may also support health prevention efforts, increase women’s empowerment, and improve gender equity in household decision-making processes [10]. Existing literature on whether MFIs are successful in ensuring sustainable livelihoods, however, is mixed. A series of randomised controlled trials found little impact of one popular MFI, microcredit, on household income and consumption [11]. Other studies of interventions that combine cash transfers or productive assets with business and financial training, meanwhile, observed sustained improvements in economic outcomes [12,13]. Many of these studies found substantial heterogeneity in programme outcomes, suggesting that social capital and initial resources may help determine whether MFI programmes achieve their objectives [13–16].

Together, this research suggests that the success of MFI programmes in achieving sustainable livelihoods depends on them being designed with key participant and household characteristics in mind. However, there is little research on perceived barriers and facilitators of programme success among MFI participants, how MFI programme design accounts for these perceptions, and the collective implications of programme barriers and facilitators for well-being. To address this gap in the literature, we sought to understand participants’ perceptions of the barriers and facilitators to a poultry microenterprise intervention, a specific MFI that has been applied in many low-income settings [17,18]. Following a longitudinal, independent poultry rearing intervention conducted with women in rural, southwestern Uganda, we conducted qualitative exit interviews to examine participants’ perceptions of the project, particularly in relation to income, health, education, and quality of life over time. We also explored perceptions of the impact of the poultry microenterprise intervention on enabling sustainable livelihoods for the participants, their families, and their community.

## METHODS

### Setting

We conducted interviews in Nyakabare Parish with women who had previously participated in a poultry microenterprise demonstration project. Nyakabare Parish, Rwampara District, lies on the outskirts of Mbarara Town, approximately 260 km southwest of Kampala, the capital of Uganda. Most parish residents work in subsistence agriculture, and poverty and food and water insecurity are pervasive [19–22]. Savings and credit cooperatives and loans are common in this setting [23,24], and many individuals request informal loans from friends, family, and neighbours during times of economic strain. Gender dynamics in this context limit women’s mobility, and most women primarily assume unpaid family responsibilities such as household work and care roles for children, the sick, and the elderly [25].

### Poultry microenterprise demonstration project

**Figure 1:**
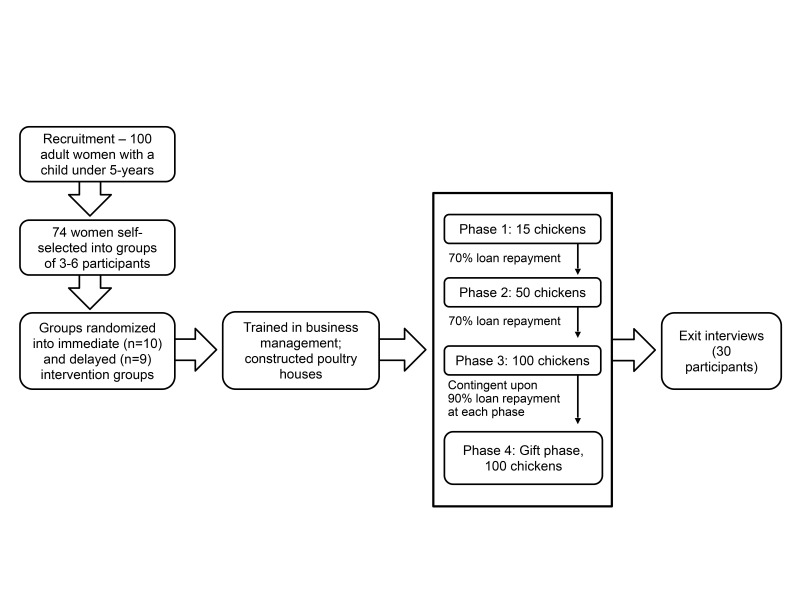
Flowchart of poultry microenterprise demonstration project.

A poultry microenterprise demonstration project, focussed on improving economic, health, and social outcomes of participating women and their children, was implemented with parish residents from December 2016 to December 2018. Embedded in an existing longitudinal population-based study, the poultry microenterprise intervention involved the recruitment of 100 adult women with at least one child under five years old residing in their household. These women were required to self-select into a group of 3–6 women to foster group accountability among members. Given pervasive gendered power dynamics and husbands’ reluctance for their wives to enrol in the project, our team struggled to recruit all 100 participants. Ultimately, we enrolled 92 women in the intervention, who then formed 19 groups. Due to resource constraints, we randomly allocated the groups to either the immediate intervention group (10 groups) or the delayed intervention group (9 groups) (**Figure 1**). Participants built chicken coops on their property and attended educational workshops to learn how to rear and sell poultry for meat. Topics included regulating the temperature of the chicken coop, and feeding, watering, vaccinating, treating, and selling the chickens. The participants also participated in bookkeeping/finance exercises to develop business skills and encourage successful marketing and loan repayment.

After completing training, participants received their first in-kind microloan: 15-day-old chicks and other materials (mats, coffee husks, stoves, charcoal, drinkers, feed, vaccines, medicines) to begin a poultry business (phase 1). Three months later, participants who advanced to phase 2 received 50 chicks; after another three months, participants who advanced to phase 3 received 100 chicks. Participants advanced if they successfully paid back at least 70% of their loan from the previous phase. If able to repay at least 90% of their loan at each phase, participants received a ‘gift’ of 100 birds, feed, and medicine as start-up capital to continue their poultry rearing business (phase 4). The delayed intervention groups went through these procedures 24 months after the immediate intervention groups.

### Qualitative exit interviews

About nine months following the completion of the four-phase poultry microenterprise intervention, bachelor’s- and master’s-level research staff (JK, AK, EBN) made field visits to conduct face-to-face in-depth interviews with intervention participants. These visits occurred from September to December 2019. Although the research staff conducting interviews did not implement the intervention, they were affiliated with the implementation team. Further, they were all research assistants on the aforementioned population-based longitudinal survey conducted in the same setting.

We purposely selected participants from the immediate and delayed intervention groups. Women were eligible for interviews if they had participated in the demonstration project and were available to complete the interview in or near their homes at the agreed-upon appointment time. Recruitment was halted once the research team reached thematic saturation, *i.e.* when, while iteratively reviewing transcripts and developing the codebook, the team determined that they were no longer eliciting new themes or subthemes.

Thirty women participated in exit interviews and were included in the analysis. No women declined participation. The interviews lasted approximately 30–60 minutes, and the research staff took field notes during and after the interviews. Each interview took place in a private location near the participants’ houses; no other individuals were in earshot.

We also collected basic demographic characteristics during the interviews. For this purpose, the research team asked women to rate their household wealth in relation to others in the village (better off, average, worse off) [26]. We administered standardised scales to assess food insecurity [20] and water insecurity [19]. Finally, women who were married or cohabiting reported how much they trusted their partner with money on a four-point Likert-type scale.

During the exit interviews, the research team asked open-ended questions according to a semi-structured interview guide, with follow-up probes. The team first asked participants to describe their experiences in the project and then elicited their perceptions of the benefits of the project across various facets of life (*e.g.* health, education, quality of life), facilitators and challenges of the project, and barriers to completing all phases. Finally, participants were asked to explain the circumstances that enabled or hindered the development of sustainable poultry rearing businesses.

### Qualitative data analysis

Interviews were conducted in the local language (Runyankore) and audio-recorded; they were simultaneously translated and transcribed as they were being completed. Following a qualitative training workshops led by BFOB, the team used an inductive qualitative content analysis approach to analyse the data [27]. Members of the research team (JK, AK, EBN, BFOB, CB, ENS) collaboratively reviewed transcripts and iteratively developed a codebook outlining initial themes and subthemes. All interview transcripts were double-coded in Dedoose (JK, CB) using the codebook developed by the team.

## RESULTS

We interviewed 15 women from the immediate intervention group and 15 from the delayed intervention group. Overall, 20 of the 30 interview participants reached phase 4 and received the gift of 100 chickens. The other ten participants either dropped out during an earlier phase or were unable to pay back at least 90% of their loan after each phase. Across all 92 women who participated in the poultry microenterprise demonstration project, 47 reached phase 4.

The average age across both groups was 40.1 years (standard deviation = 8.42). Twenty-seven of the 30 women were married, and half of these women trusted their partner with money ‘often’ or ‘all the time’. Just under half of the participants were food secure (n/N = 14/30, 46.7%), while most were water secure (n/N = 19/30, 63.3%). Two-thirds of the participants (n/N = 20/30, 66.7%) perceived that they had about average wealth compared to other parish residents (**Table 1**).

**Table 1:** Characteristics of exit interview participants stratified by immediate and delayed intervention group*

	Intervention group		
	**Immediate (n = 15)**	**Delayed (n = 15)**	**Total (n = 30)**
**Age in years, mean**	38.7 (6.78)	41.6 (9.83)	40.1 (8.42)
**Married**			
Yes	13 (86.7)	14 (93.3)	27 (90)
No	2 (13.3)	1 (6.67)	3 (10)
**Trust partner with money**			
Never	0 (0)	6 (42.9)	6 (22.2)
Once in a while	3 (23.1)	3 (21.4)	6 (22.2)
Often	4 (30.8)	2 (14.3)	6 (22.2)
All the time	6 (46.2)	3 (21.4)	9 (33.3)
**Education**			
Completed primary school	8 (53.3)	9 (60)	17 (56.7)
Did not complete primary school	7 (46.7)	6 (40)	13 (43.3)
**Self-perceived wealth**			
Better off	3 (20)	3 (20)	6 (20)
Average	9 (60)	11 (73.3)	20 (66.7)
Worse off	3 (20)	1 (6.67)	4 (13.3)
**Household food insecurity**			
Food secure	6 (40)	8 (53.3)	14 (46.7)
Mild food insecurity	2 (13.3)	1 (6.67)	3 (10)
Moderate food insecurity	7 (46.7)	5 (33.3)	12 (40)
Severe food insecurity	0 (0)	1 (6.67)	1 (3.33)
**Household water insecurity**			
Water secure	10 (66.7)	9 (60)	19 (63.3)
Mild water insecurity	1 (6.67)	2 (13.3)	3 (10)
Moderate food insecurity	3 (20)	2 (13.3)	5 (16.7)
Severe food insecurity	1 (6.67)	2 (13.3)	3 (10)

*Presented as n (%) unless specified otherwise. Figures do not add to 100% due to rounding.

**Figure 2:**
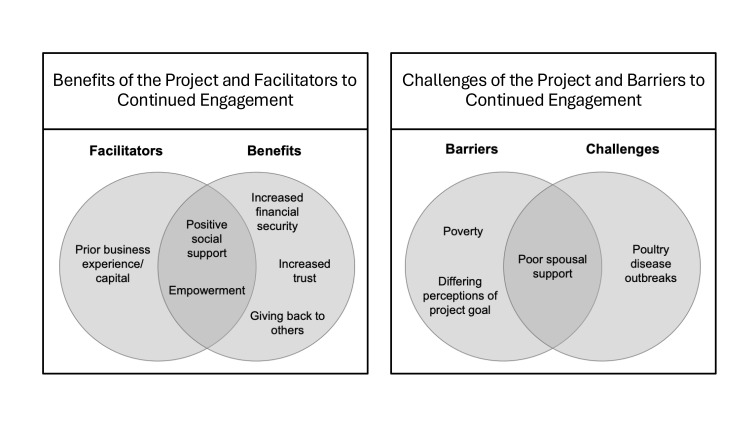
Benefits and challenges of the project and facilitators and barriers to continued engagement.

During interviews, participants outlined multiple benefits and challenges of the project, as well as facilitators and barriers to continued engagement (**Figure 2**). Some of the themes overlapped as both benefits/facilitators or challenges/barriers. We discuss them in depth below.

### Benefits of the project and facilitators to continued engagement

#### Increased financial security

The participants discussed several instrumental and interpersonal benefits of engaging in the poultry microenterprise intervention. Many noted that they were able to use profits from the project to address basic household needs. For example, they described using profits to pay for school fees, medical bills, and home renovations. One woman elaborated on some of the essential items she could buy due to her participation: ‘I could use the money to buy chairs for my house or buy other items that were lacking in my home’ (participant 1, 31-year-old woman, immediate intervention group). The chickens provided them with a source of financial security, thus enabling participants to address their basic needs. The same participant described, ‘We would look at the chickens as a source of security, just in case you got any problems that required spending some money. So, we were well off financially (…)’.

#### Increased trust

Along with increased financial security, the participants also discussed increased trust from other community members. Because the participants had a steady income from their poultry businesses, they felt other community members were more willing to give them loans:

Or when I asked to borrow money from someone, they could give it to us because they knew that once you had chickens you could be able to pay back after selling the chickens. So, the chicken project made it easy for us to relate with other people when it came to getting loans from other people. *– Participant 1, 31-year-old woman, immediate intervention group.*

#### Prior business experience and capital

While participants widely viewed the financial and interpersonal benefits of the project as facilitators of their ability to move through the phases, some of the women were more successful in completing all of the phases and securing sustainable livelihoods than others. Entrepreneurial women with other income-generating side projects and women with more capital at the start of the intervention reported being better able to save their profits and proceed through all project phases. For example, one participant reported that she has continued to rear chickens since the project concluded, alongside her other business activities: ‘I go for gardening, weeding, and also this [hotel] business, but I make sure that my chickens are [still] given time’ (participant 12, 35-year-old woman, immediate intervention group).

#### Positive social support

Besides the project’s financial benefits, women described how the support they had received from other group members, their partners, and the project demonstration team also helped them stay engaged and achieve success. One woman described how the project improved her communication with her husband, as they worked together on this new business venture:

Truth be told, we did everything together and completed successfully without any quarrels resulting from the chickens because he would take them to sell and bring the money as it is, and we plan for that money, and get what we had intended to get from that money. *– Participant 2, 43-year-old woman, immediate intervention group.*

Other participants reiterated the importance of spousal support, listing the many ways in which their partners supported them in their project:

(…) seriously, [my husband] could help me (…) he could give drinking water to the birds (…) he could put up the fire for them (…) he could see which chicken was sick (…) And when the birds were ready for selling, he used his efforts to take the birds and sell them, and he could bank the money because I trusted him. *– Participant 3, 50-year-old woman, immediate intervention group.*

Another stressed, ‘my husband was there for me (…) [we] worked together in selling the chickens’ (participant 4, 34-year-old woman, delayed intervention group).

While many women received support from their partners to engage in their poultry businesses, many also emphasised how support from the project field officers helped them move through the project phases. In particular, field officers’ advice on how to handle poultry diseases and loan repayments provided participants with the information and tools to successfully navigate challenges:

Those people were with us in everything we did because they were the people who used to advise us. When one would get hardships in anything, you would call them on the phone, and they would come and counsel you about what was becoming hard. *– Participant 2, 43-year-old woman, immediate intervention group.*

#### Empowerment

A central theme of the interviews was that the women shared how their poultry businesses made them feel empowered. One woman expressed that she experienced an increased sense of worth through her participation. Describing feelings of empowerment, she stated, ‘you get more added unto you’ (participant 2, 43-year-old woman, immediate intervention group)

Another participant shared how this new sense of empowerment motivated her to continue independent ventures to help support her family:

(…) I was motivated so much to learn that a woman can also work hard and make money on her own. This is very important because it gives a woman a platform to do anything, even if it is small, to make sure one can earn a living as a woman and support our families. *– Participant 5, 44-year-old woman, immediate intervention group.*

Women who felt more empowered, hopeful, and determined to succeed in the project were more successful in completing all its phases. Furthermore, participants in the delayed intervention group described heightened empowerment and confidence in their ability to manage their businesses since they were able to observe and learn from the challenges faced by participants in the immediate intervention group.

After participating in the project, several women continued to appreciate the project for the skills they gained. These skills empowered them to become more self-reliant and financially independent from their husbands. As one participant stated, ‘We benefited a lot in terms of skills. We are no longer *bareera* [uninformed/unfamiliar], we are no longer ignorant, we can survive on our own, not waiting for a man to earn for us’ (participant 5, 44-year-old woman, immediate intervention group).

#### Giving back to others

Besides recognising their increased self-reliance, many participants appreciated the opportunity to advise others and give back to their communities. With newfound confidence in their skills, they could instruct their friends and neighbours in developing their own poultry businesses. As one participant described, ‘you can find me being someone who can give advice to people on something that I previously never knew about’ (participant 1, 31-year-old woman, immediate intervention group). The participants who fully embraced the project as a business opportunity to ensure a sustainable livelihood for their families described being more successful completing the phases, with a few participants continuing their ventures past the gift phase.

### Challenges of the project and barriers to continued engagement

#### Poverty

Although participants described the benefits and facilitators of the intervention that helped them be successful, they also expressed challenges that hindered them from continuing past the gift phase and developing sustainable livelihoods. Pervasive poverty stood as a major barrier, with many participants describing using the entirety of their profits to fulfil family needs, such as paying for school fees, house repairs, and other necessities. For example, one woman described how another participant could not continue with the project because she used all the funds from her business to pay the school fees for her child:

But as for her, after selling the chickens for the gift phase, she had a child in secondary school, and she paid all the money for school dues but did not continue with the chickens. However, she loved chickens. But because the child was in [Secondary 4], registering for the final exams, I think all the money was spent in doing that. *– Participant 6, 37-year-old woman, delayed intervention group.*

Similarly, another woman described how paying school fees with her profits prevented her from continuing with the business venture:

I failed to get more money because of school fees for my children, [laughing] thus I could not continue. I would tell my husband, ‘let me put more chickens.’ And he would say, ‘you see how I am. I have no more money after paying school fees.’ And when we included construction in our programmes, his situation completely worsened for money. So, I could not put pressure on my husband to give me money to buy chickens. I had to leave the project. *– Participant 7, 43-year-old woman, immediate intervention group.*

Although several participants appreciated the demonstration project for providing them with the financial security to afford necessities for their households, buying these necessities with their profits also limited their ability to continue with the project on their own. One participant described:

I bought a new metallic door because the wooden one that I had was eaten by the termites and it was as if I used to not close the windows. They were affected by the termites. But what you are seeing now is what I got from the gift phase of the chickens that you gave me. That is how I spent the profit that I made, and what affected my continuity with the [project] on my own. Because the little money that I made was spent on those things. *– Participant 2, 43-year-old woman, immediate intervention group.*

#### Poultry disease outbreaks

Some participants also struggled with their chickens contracting diseases and dying. As one explained:

We had a challenge of losing chickens because of the diseases. This was mostly during the last gift phase. Some of us were complaining about this. We were wondering why our chickens in the gift phase were dying more compared to the other phases when we were paying back the loans. That gave us a hard time. *– Participant 8, 33-year-old woman, delayed intervention group.*

Since these participants already felt financial strain due to economic insecurity and demands on their profits, many worried that continuing with the business past the gift phase might lead to financial losses, particularly if their chickens died of disease before they were able to take them to market.

#### Poor spousal support

While some participants highlighted support from their partners as a facilitator to their success in the project, other participants described needing to drop out early due to poor spousal support. One woman described how her partner’s lack of engagement served as a barrier to her engagement, ‘For sure, there was nothing he was involved in. I cannot tell lies to you. Let it be raising them, selling them…he did not help me at all on anything regarding poultry keeping’ (participant 9, 50-year-old woman, delayed intervention group). Others explained how some of their group members were not successful in the project because their husbands did not want them to continue with it, either because they did not want to help drive the chickens to market or because they did not trust the project staff. For example, one participant explained, ‘some members got challenges whereby their husbands stopped them from rearing the chickens and they had to stop’ (participant 4, 34-year-old woman, immediate intervention group).

One participant even alluded that her husband stole some of her profits. Although he helped sell the chickens at the market, he would bring back less money than anticipated based on the projected sales:

He used to tell me that he sold and got such and such amount, but for sure, after calculation, the money would not total the number of chickens taken. I realised that I am going to have so many debts, yet [the team] who brings the chickens needs to be paid. I thought, ‘what I will tell them?’ So I said, since I have failed, let me say the truth. And I had to pay some money that I managed to pay. And I dropped out to allow my colleagues to continue. *– Participant 10, 40-year-woman, delayed intervention group).*

#### Differing perceptions of project goal

Finally, while the long-term goals of the demonstration project were to empower women and ensure sustainable livelihoods for the participants and their families (*i.e.* through continuing their chicken businesses or engaging in other income-generating activities with proceeds from the chickens), many participants held different perceptions of the project goals. Many participants, particularly those who had not previously had their own businesses, perceived that they had succeeded in the project once they received the gift phase. Instead of viewing the final gift of 100 chickens as start-up capital to put toward continuing their business ventures, these women saw reaching the gift phase as the end goal; they believed that after selling these chickens and using the profits on household necessities, they were done with the project.

### Feasibility of the demonstration project in creating sustainable livelihoods

Six (20%) of the exit interview participants continued rearing chickens after the gift phase, including one woman who dropped out after phase 1 to start her business on her own timeline and rear more chickens at a time. Additionally, a few parish residents who were not involved in the intervention started their own poultry businesses after observing their friends and neighbours successfully rear and sell chickens for supplemental income. Women who continued past the end of the demonstration project generally considered poultry rearing to be ‘a project that makes quick money’ (participant 11, 43-year-old woman, immediate intervention group), had the support of their spouses and family, and had supplemental income to stock chickens past the gift phase (*i.e.* from entrepreneurial ventures, formal employment, and small-scale commercial farming (animal husbandry; growing maize, millet, and matooke, a local food and cash crop)).

One participant who successfully continued her poultry business described how the venture helped improve her financial situation, including increasing her family’s access to food: [Chicken rearing] has really helped us. For example, my husband these days has not been having a job, but any time I realise that the children have no food, I decide to go to the bank, withdraw some money, and buy some maize flour. And when we later get the money, we take it back and save. When we decide to go and book the chickens, the money is on the account. *– Participant 6, 37-year-old woman, delayed intervention group.*

While some women were successfully able to complete all four phases and continue their poultry rearing businesses, poverty, spousal problems, and varied interpretations of project ‘success’ prevented most women from continuing past the gift phase. However, women who did not continue their business past the gift phase and participants who dropped out during earlier phases were still excited about the project and interested in participating again if the opportunity arose. As one individual described, ‘we’re praying and saying that in the future if [the team] brings another project that could give us 100 chickens or even more (…) for us we are ready to again participate in that project’ (participant 3, 50-year-old woman, immediate intervention group).

## DISCUSSION

In this qualitative study among women participating in a poultry microenterprise demonstration project in rural Uganda, participants described both its benefits and challenges. Many stressed how the project supported their family’s finances and gave them a sense of empowerment, noting how instrumental and interpersonal support helped them navigate through the intervention phases. Yet, despite its benefits, most women did not continue with their business after the formal completion of the project. While a few participants, particularly those with supplemental income and strong spousal support, successfully continued rearing chickens to ensure sustainable livelihoods for their families, most cited barriers that hindered them from developing independent poultry-rearing businesses.

In the context of pervasive poverty, financial demands limited women from continuing with the project or sustaining a business past the intervention period. While these women could use some profits from the intervention to cover school fees and other household needs, paying for these necessities prevented them from saving money from the project and limited opportunities for longer-term engagement. Spousal mistrust and fears of poultry disease outbreaks provided further financial stress and inhibited opportunities for developing sustainable livelihoods that are resilient to external stressors. These findings are consistent with the growing body of empirical research documenting heterogeneous returns to MFI programmes [15]. For example, a prior review highlighted how individual-, household-, and community-level structures may limit the success of some women in entrepreneurial pursuits [28]. The present data identify a larger range of potential barriers and facilitators of programme success than those considered in much of the research on MFIs to date [11,13–16].

Our findings have important implications for the design, implementation, and evaluation of future microenterprise interventions. When considering how to best develop sustainable livelihoods through similar programmes, it is important to consider the sustainability of the MFIs themselves. It is also critical that researchers and programme administrators consider the local context in collaboration with community members [28]. While microfinance interventions have supported the development of sustainable livelihoods in other settings [12,13], differences across contexts may impact the sustainability of business ventures. By exploring cultural norms and attitudes through collaborations with local partners, community engagement meetings [29], and formative needs assessments [30], programmes can be adapted to best support sustainability in line with the context.

While participants enjoyed the intervention’s group work component, sustainability was hindered by poverty and financial needs. Future interventions may consider enabling compulsory savings mechanisms within the group work structure. Cooperative savings groups and social capital both contribute to poverty reduction in Africa [24,31]. Encouraging cooperative savings within the group format may help prevent participants from spending all their profits at once, at the expense of saving money to continue their businesses. For example, each month, group members might be expected to contribute a certain percentage of their profits into its savings account. Most of the savings pool would then go toward buying new chickens and supplies to support members’ businesses, while a smaller percentage would be kept as a group fund for emergency loans. Such mechanisms would provide group members with accountability toward the project while limiting interruptions by external financial stressors. To further support participants in saving profits from their business ventures, initial project training may be adapted to emphasise the importance of savings.

While partner support benefited some women in their businesses, a lack thereof limited others from being able to continue poultry rearing. This reflects evidence from microfinance interventions in Ghana and South Africa, in which some projects led to worsening family relations [32]. By actively involving participants’ spouses or partners in training and throughout the intervention, interventions may increase spousal collaboration and the development of sustainable livelihoods. If men participate in training sessions with their partners and attend training on how to support their partners themselves, they may develop a small sense of ownership and a greater commitment to the sustainability of the project. Particularly in Nyakabare Parish, gender unequal norms lead to men acting as the primary decision-makers in the household. Engaging them in the project and emphasising how the business can be a team effort may help support savings and investment in project success.

### Limitations

First, the participants knew the research staff conducting interviews and had established ties with the team that implemented the poultry microenterprise demonstration project. Thus, social desirability bias may have impacted their responses. Second, given the qualitative methodology used here, whereby only a select subset of participants participated in exit interviews, these results may not generalise to the entire group of women who participated in the intervention. Furthermore, microenterprise interventions in other settings may experience other barriers and facilitators to sustainability. However, financial and social concerns are likely to be present in other settings in Africa. These findings and recommendations may be helpful in supporting projects in other settings to frame research aims and navigate challenges.

## CONCLUSIONS

Although participants reported several benefits from the poultry microenterprise demonstration project, few continued rearing chickens past the final study phase. They reported financial and other barriers that limited them from developing long-term businesses to secure sustainable livelihoods. Since the intervention proved difficult to operationalise in this environment, future microfinance and agriculture interventions should work with participants to understand obstacles to sustainability. Besides maintaining group work and field staff involvement, providing more chickens to enhance economies of scale, instituting compulsory savings mechanisms over immediate consumption, and targeting entrepreneurial individuals could go a long way to make poultry microenterprise more feasible in this setting. By addressing these challenges, research and programmatic institutions can maximise the impact of investment in such interventions and ensure longer-term economic benefit for participants.
